# On the Effects of the Lateral Strains on the Fiber Bragg Grating Response

**DOI:** 10.3390/s130202631

**Published:** 2013-02-21

**Authors:** Marco Lai, Dimitris Karalekas, John Botsis

**Affiliations:** 1 École Polytechnique Fédérale de Lausanne (EPFL), LMAF-IGM-STI, CH-1015 Lausanne, Switzerland; E-Mail: marco.lai@epfl.ch; 2 Laboratory of Advanced Manufacturing Technologies and Testing, University of Piraeus, GR-18534 Piraeus, Greece; E-Mail: dkara@unipi.gr

**Keywords:** fiber optic sensor, optomechanical constant, biaxial loading, hydrostatic pressure, epoxy, carbon/epoxy

## Abstract

In this paper, a combined experimental-numerical based work was undertaken to investigate the Bragg wavelength shift response of an embedded FBG sensor when subjected to different conditions of multi-axial loading (deformation). The following cases are examined: (a) when an isotropic host material with no constrains on planes normal to the embedded sensor's axis is biaxially loaded, (b) when the same isotropic host material is subjected to hydrostatic pressure and (c) when the hydrostatically loaded host material is an anisotropic one, as in the case of a composite material, where the optical fiber is embedded along the reinforcing fibers. The comparison of the experimental results and the finite element simulations shows that, when the axial strain on the FBG sensor is the dominant component, the standard wavelength-shift strain relation can be used even if large lateral strains apply on the sensor. However when this is not the case, large errors may be introduced in the conversion of the wavelength to axial strains on the fiber. This situation arises when the FBG is placed parallel to high modulus reinforcing fibers of a polymer composite.

## 1. Introduction

Monitoring the internal strain state of fiber-reinforced polymer materials has become an important issue in many performance demanding engineering applications (e.g., aerospace, naval, civil). Fiber Bragg grating (FBG)-based sensors are excellent candidates for that purpose as they combine many advantages over conventional sensors (e.g., strain gages). Such advantages are, for example, their small size, immunity to electromagnetic interference, multiplexing capabilities, resistance to corrosion and self-referencing ability, together with an often linear response that is encoded in the change of their reflected resonance wavelength. The possibility of embedding optical fibers in anisotropic materials has already been demonstrated in numerous investigations [1–4]. Most of the work published so far has focused on axial load and temperature variations in addition to out-of-plane strain, which is a key parameter in the structural integrity of anisotropic materials [5–7]. In recent years substantial research efforts have also attempted to develop multi-parameter FBG sensors. Several reports have described the characterization of gratings subjected to transversal strains which can induce birefringence to the optical fiber and therefore split the reflected Bragg peak into separate ones, each relative to polarization axis [8].

Although the FBG sensor provides the benefits of *in situ*, real-time monitoring, it can also produce ambiguous results due to its sensitivity to all strain components. Understanding the strain state on an embedded optical fiber is not straightforward. Due to multi-axial sensitivity of a fiber Bragg grating sensor there are cases where a simplification of the governing optomechanical equations based on the assumption that the transverse strains are related to the longitudinal strain by the Poisson's ratio of the glass fiber can result in an incorrect interpretation of a single wavelength shift. For instance, in the case of the thermal expansion of an anisotropic material like a carbon-polymer composite, the transverse strains may become large relative to the longitudinal strain since the coefficient of thermal expansion of the fiber is close to zero. As a result the above assumed optomechanics theory relationship (described in Section 2) cannot be applied. This can lead to significant errors in the axial strains measured by the sensor. Such a strain state could be easily overlooked since no peak split in the recorded wavelength could appear.

Furthermore, high relative transverse loads may develop during the potential application of FBG sensors in high pressure environments found in well and deepwater applications. For instance, real time monitoring of fluid pressure in bare-holes is crucial for the control of oil production in offshore oil fields [9]. A resolution of about 10 kPa (0.1 bar) or better is required at pressure levels up to 100 MPa (1,000 bar) [10]. Consequently, measurement reliability is essential to realizing the full benefits offered by the deployment of FBG sensors for monitoring purposes.

This paper presents the characterization of a typical FBG sensor in applied strain fields where the transverse strains around the fiber are equal or significantly higher than the one along the fiber direction. Experimental and numerical results are presented.

## 2. Optomechanics Principles

**Figure 1.**
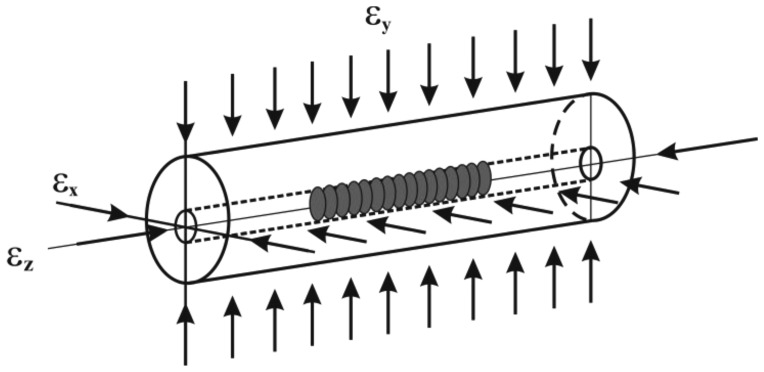
Schematic of an optical fiber with an FBG sensor subjected to strains.

When a Bragg grating, written on the core of a conventional single mode (SM) optical fiber, is subjected to strain along its *z* axis, (see Figure 1), the wavelength strain relation is expressed as follows ([11], and references therein):

(1) $$\frac{\Delta \lambda_{B}}{\lambda_{B0}}={ɛ}_{z}-\frac{n_{\text{eff}}^{2}}{2}\left[p_{11}{ɛ}_{x}+p_{12}\left({ɛ}_{y}+{ɛ}_{z}\right)\right]$$

Here *ε_x_* = *ε_y_,ε_z_* are the applied homogeneous strains on the fiber (Figure 1); *λ_B_*_0_, *λ_B_* are the reference and shifted (*i.e.*, after straining) Bragg wavelengths, respectively; *n_eff_* is the effective refractive index of the core of the unstressed fiber; *p*_11_ and *p*_12_ are the Pockel's strain-optic coefficients. If a temperature difference is applied on the fiber, a compensation to the wavelength changes should be added to the right hand side of Equation (1).

In cases where a three dimensional strain field with *ε_z_* ≠ *ε_x_* ≠ *ε_y_* is present at the location of the grating, a non-negligible sensor response to transverse strains must be accounted for. When two different transverse strains, namely *ε_x_* and *ε_y_*, are applied to a FBG the reflective index changes and splits into two values. This appearance of birefringence leads to the separation of the single reflected Bragg peak into two distinct ones [8,12,13]. The wavelength shifts are given by:

(2) $$\frac{\Delta \lambda_{B,x}}{\lambda_{B0}}={ɛ}_{z}-\frac{n_{\text{eff}}^{2}}{2}\left[p_{11}{ɛ}_{x}+p_{12}\left({ɛ}_{y}+{ɛ}_{z}\right)\right];\frac{\Delta \lambda_{B,y}}{\lambda_{B0}}={ɛ}_{z}-\frac{n_{\text{eff}}^{2}}{2}\left[p_{11}{ɛ}_{y}+p_{12}\left({ɛ}_{y}+{ɛ}_{z}\right)\right]$$

where *ε_z_*, *ε_x_ε_y_* are the principal homogeneous strains in the core of the SM fiber, directed along the axial and the two polarization axes. It is noted that the peak separation is proportional to the difference between the transversal strains *ε_x_* and *ε_y_* only, easily expressed as:

(3) $$\frac{\Delta \lambda_{B,y}-\Delta \lambda_{B,x}}{\lambda_{B0}}=\frac{n_{\text{eff}}^{2}}{2}\left(p_{12}-p_{11}\right)\left({ɛ}_{y}-{ɛ}_{x}\right)$$

In the case of equal transverse strains *ε_x_* and *ε_y_*, the previously discussed birefringence is removed and Equations (2) are reduced to Equation (1).

In most cases of practical interest, the FBG is subjected to axisymmetric homogeneous strains, *i.e.*, *ε_x_* = *ε_y_*. Assuming further that the lateral strains are related to the axial one by a standard Poisson relation *ε_x_* = *ε_y_* = −*ν_f_ε_z_*, Equation (1) is reduced to the following simplified relation:

(4a) $$\frac{\Delta \lambda_{B}}{\lambda_{B0}}=(1-p_{e}){ɛ}_{z}$$

where:

(4b) $$p_{e}=\frac{n_{\text{eff}}^{2}}{2}\left[p_{12}-v_{f}(p_{11}+p_{12})\right]$$

is the effective optomechanical constant and *ν_f_* is the fiber's Poisson ratio. Equation (4a) has been used by several researchers to measure strains in various experimental configurations by monitoring the wavelength shift *Δλ_B_* since *λ_B_*_0_,*p_e_* are known parameters for a given sensor. While *λ_B_*_0_ is known from the FBG inscription, *p_e_* is calculated by inserting the values of the parameters. In this work, these parameters are taken as follows: *n_eff_* = 1.468 (typical value for an optical fiber, *i.e.*, the Corning SMF-28™ fiber) and *p*_11_ = 0.121, *p*_12_ = 0.270 for silica [14]. As for Poisson's ratio, it was assumed as *ν_f_* = 0.19. Accordingly, it is found that *p_e_* = 0.2108. Note that *p_e_* can also be measured experimentally using Equation (4a) at ambient temperature since the fiber properties can vary according to the manufacturer.

Implicit to using Equation (4a) for an embedded fiber is the assumption that the axial strain on the sensor is the dominant one and the lateral ones are simply due to Poisson as initially proposed by Butter and Hocker [15]. However, if the experimental conditions are such that the axial component is not the dominant one and the lateral strains are significant, use of Equation (4a) is not realistic.

To examine the effects of relatively large lateral strains on the FBG sensor, the transversal strains are assumed homogeneous and equal (*ε_x_* = *ε_y_*) but they are not related to *ε_z_* by a simple Poisson's relation but by a *biaxiality ratio* defined as:

(5) $$\frac{{ɛ}_{x}}{{ɛ}_{z}}=B$$

**Figure 2.**
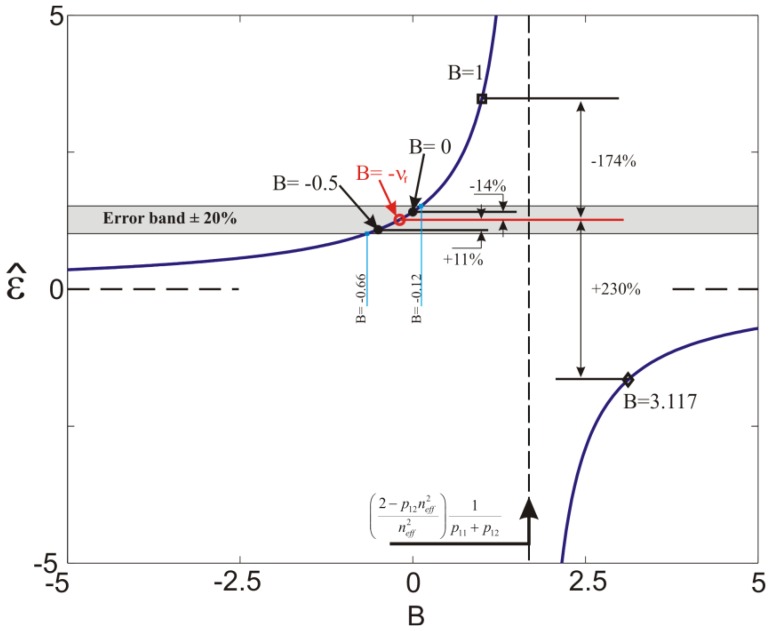
Evolution of normalized axial strains according to Equation (7). See text for details.

Thus Equation (4a) can be expressed as:

(6a) $$\frac{\Delta \lambda_{B}}{\lambda_{B0}}=\left(1-p_{e}^{\text{eff}}\right){ɛ}_{z}$$

where:

(6b) $$p_{e}^{\text{eff}}=\frac{n_{\text{eff}}^{2}}{2}\left[p_{12}+B(p_{11}+p_{12})\right]$$

is now an effective photo-elastic coefficient. To illustrate the effects of *B* on $p_{e}^{\text{eff}}$ Equation (6a) is rewritten as:

(7) $$\hat{ɛ}=\frac{{ɛ}_{z}\lambda_{B0}}{\Delta \lambda_{B0}}=\frac{1}{1-p_{e}^{\text{eff}}(B)}$$

where *ε̂* is a normalized parameter introduced for convenience. The evolution of the right hand side of Equation (7) as function of _B_ is shown in Figure 2. Also shown in Figure 2 are the two asymptotes exhibited by Equation (7): (a) a vertical one at $p_{e}^{\text{eff}}(B)=1$ for $B=\left(\left(2-p_{12}n_{\text{eff}}^{2}\right)/n_{\text{eff}}^{2}\right)/\left(p_{11}+p_{12}\right)$ and (b) a horizontal one at $1/\left(1-p_{e}^{\text{eff}}(B)\right)=0$ for $p_{e}^{\text{eff}}(B)=\pm \infty$. The grey band corresponds to an error of ±20% from the strain value given by Equation (7) with *B* = −*ν_f_*. This is the case where Equations (4) and (7) give the same results. The strain value, at *B* = 1 corresponds to the FBG sensor subjected to hydrostatic pressure and the strain at *B*_= 3.1170_ to a uniaxial composite under hydrostatic pressure where an FBG sensor is embedded parallel to the reinforcing fibers. These two cases are discussed in the next sections of the paper.

Note that the importance of the lateral strains on the response of the FBG is discussed in [16]. The authors define an effective Poisson's ratio (similar to parameter *B*, Equation (5)) and examine numerically the effects of the FBG's coating modulus, the host's material properties and thickness, as well as the length of the sensor on the sensor's response. Although the numerical simulations shed light on the influence of these parameters on the effectiveness of the sensor under uniform applied thermal strains, the results are not verified experimentally. In the present work, a combined experimental-numerical approach is undertaken to explicitly and quantitatively assess the influence of the lateral strains on the sensor's response (*i.e.*, the limits of Equation (4)). Experimental configurations including isotropic and anisotropic host materials are investigated without the sensor's coating since the latter can limit the efficiency of the strain transfer between the sensor and the host material.

## 3. Materials and Methods

To examine the effects of relatively high lateral loads on the FBG strain response, two conditions were realised experimentally. The first one was a biaxial loading on an epoxy block and the second one a hydrostatic pressure applied on the bare fiber, epoxy cylinder and uniaxial carbon epoxy plate. In all experiments SM optical fibers, with 125/9 μm cladding/core diameters and a 5 mm FBG sensor, without any coating, operating at the wavelength of 1,550 nm, were used. The Bragg wavelength peak shifts were recorded using a Micron Optics sm130 optical sensor interrogator. Note that the uncertainty in the wavelength measurement was 14pm which, according to the value of photoelastic constant, resulted in an uncertainty in strains between 11 (for *p_e_* = 0.2108) and 30με (for $p_{e}^{\text{eff}}=0.7122$). These uncertainties are not shown in the figures with the results for better clarity. All experiments were simulated using linear elasticity based finite element (FE) models in the Abaqus solver v6.8.

### 3.1. Biaxial Loading

For the biaxial loading, an epoxy block with dimensions 12 mm × 12 mm × 38 mm was prepared with the sensor at its center. The material was a mixture of the DER330, DER732 resins and the DEH26 hardener in weight proportions of 70:30:10, respectively, from Dow Chemical Company. The block was fabricated in a specially designed vertical mould that kept the optical fiber with the embedded sensor axially aligned within the resin [6]. Curing took place at 30 °C for 24 hours. An additional post-curing treatment was applied at 70 °C for 9 hours in order to increase the degree of polymerization.

**Figure 3.**
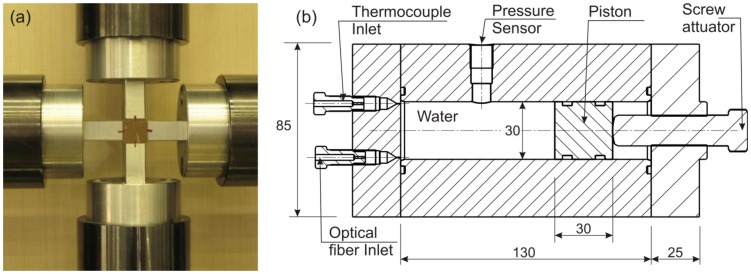
(**a**) FBG equipped specimen and loading actuators for biaxial testing, (**b**) Schematic of the pressure chamber setup (dimensions in mm).

The epoxy block was placed and tested in an Instron biaxial (cruciform) testing machine equipped with four in plane independent actuators, as shown in Figure 3(a). The testing fixtures mounted on the actuator arms provided nearly equal transversal compressive loads on the lateral sides of the specimen. Failure or slip on one side of the specimen could seriously damage the servo-hydraulic cylinders and load cells. For that reason the actual compressive area on the long sides of the specimen was reduced from 12 × 38 to 10 × 38 mm^2^. The test was performed at room temperature in load control mode in order to generate equal radial loads on the sensor and also avoid the need to account for the stiffness of loading arms. The horizontal and vertical loads were simultaneously increased from 0 to 10 kN on steps of 1kN/min and the vertical and lateral displacements imposed on the specimen were simultaneously measured using a digital image correlation (DIC) technique. These displacements were used as boundary conditions in the numerical modelling (Section 4.1).

The actuators were stopped at each applied load increment for a sufficient period of time to allow for stabilization of displacement and wavelength recordings.

### 3.2. Pressure Chamber Loading

A specially designed pressure chamber was built to apply hydrostatic pressure on the specimens. Different pressure levels were applied while simultaneously recording the water temperature with a thermocouple and the FBG wavelength peak shifts. The experimental setup is shown in Figure 3(b). To avoid any water leaks due to the relatively high pressure applied to the water, special insulating plugs were placed around the thermocouple and optical fiber at the exit points. Pressure was provided to the chamber via a piston and actuator system. By rotating the actuator, the piston was forced inside the chamber providing pressure on the specimen through distilled water inside the chamber.

In this work three experiments were carried out in the pressure chamber: (1) a bare optical fiber with the FBG sensor was tested without any special support since the surrounding water kept it straight; (2) a cylindrical epoxy specimen, of 12 mm in diameter and 40 mm in length, with a centrally located FBG was fabricated using the special casting fixture [6] with the same curing and post curing conditions as in the case of the epoxy block. The cylinder was placed in the cylindrical chamber with its axis parallel to that of the chamber; (3) a 4.2 mm thick carbon/epoxy unidirectional [0_24_] composite laminate was cured in an autoclave with conditions similar to ones reported elsewhere [17]. At the end of the curing process, a composite specimen of 28 mm in width, 36 mm in length and 4.2 mm in thickness was prepared having an FBG sensor at the specimen center. For the testing, the specimen was placed horizontally in the chamber with its long sides parallel to the chamber's axis without any special support.

### 3.3. Modeling and Data Analysis

All experimental conditions were simulated using three dimensional (3D) or axisymmetric linear elasticity based finite element (FE) models, in the Abaqus solver v6.8 with a reduced integration scheme. With these models, the strains *ε_x_,ε_y_* and *ε_z_* on the core of the fiber, as well as parameter *B* and $p_{e}^{\text{eff}}$ (Section 2) were calculated for an applied pressure of 1 MPa. Afterwards, the wavelength shifts on the FBGs sensor, in all experimental conditions, were recorded as a function of the applied hydrostatic pressure and the corresponding strains were evaluated using Equations (4) and (6). The strains for selected pressures were also extracted from the numerical models and compared with the strains given by Equations (4a) and (6a). To examine the effects of the lateral pressure only, simulations were also carried out on the bare fiber, cylindrical epoxy specimen with the embedded FBG and on the composite plate with the embedded FBG sensor.

## 4. Results and Discussion

### 4.1. Biaxial Compression: Epoxy Block

Stress analysis of the epoxy block was conducted in order to establish the degree of biaxiality exerted on the fiber when the block is subjected to equal biaxial loading. The specimen was modeled taking advantage of the specimen's three planes of symmetry planes as well as the symmetric applied loads. Thus, one eighth of the specimen was modeled with ∼19,400 3D quadratic elements. The domain was divided in 2 regions in order to model explicitly the presence the fiber along the specimen *z*-axis. The Young modulus of the epoxy in compression, measured independently, was *E_m_* = 1.9 GPa and its Poisson's ratio was assumed equal to *ν_m_* = 0.38. For the glass fiber, the corresponding properties were *E_f_* = 72 GPa and *ν_f_* = 0.19. Refinement of the mesh was also imposed around the fiber-matrix interface to avoid excessive mesh distortion. In addition, perfect adhesion of the fiber to matrix was imposed. Three types of contact conditions between the specimen and the grip were considered: (a) contact with zero coefficient of friction f = 0; (b) contacts with coefficient of friction f = 0.1; (c) contact with coefficient of friction f = 0.2.

**Figure 4.**
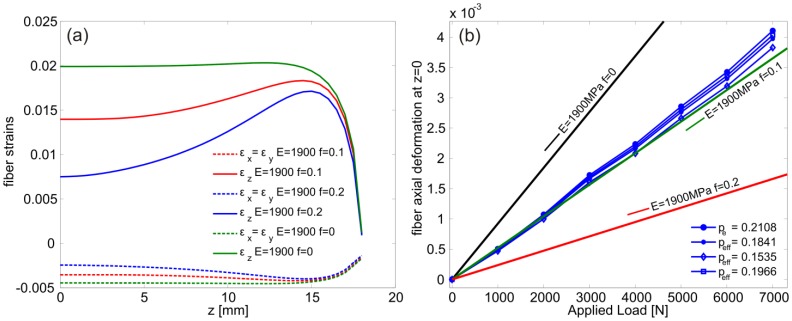
(**a**) Simulated axial and lateral deformations of the sensor in terms of *z*, (**b**) simulated axial strains at the specimen's center with different friction coefficients between epoxy block and grips compared to experimental data shown by the blue lines.

The axial and lateral deformations of the fiber as function of the specimen *z*- coordinate are reported in Figure 4(a), for a 0.1 mm displacement applied on the specimen's loading surfaces. It can be noted that the contact conditions influence remarkably the axial deformation of the fiber especially at the specimen middle plane where the FBG was placed. At this location, the biaxiality ratio *B* was calculated and used to determine the corresponding values $p_{e}^{\text{eff}}$. The following values were found: (*B_f_*_= 0_ = −0.2239, $p_{\begin{array}{cc} e & f=0 \end{array}}^{\text{eff}}=0.1966$); (*B_f_*_= 0.1_ = −0.2535, $p_{\begin{array}{cc} e & f=0.1 \end{array}}^{\text{eff}}=0.1841$), (*B_f_*_= 0.2_ =−0.3263, $p_{\begin{array}{cc} e & f=0.2 \end{array}}^{\text{eff}}=0.1535$). The three values of $p_{e}^{\text{eff}}$ were used in the Equation (6a) to convert the recorded wavelength shift to axial fiber strains which are reported, as function of the applied load, in Figure 4b. Also shown in the same figure is the fiber deformation calculated using *p_e_* = 0.2108. The results of the simulations in Figure 4(b) were obtained by imposing the corresponding displacements (to the applied loads) on the specimen's loading surfaces, measured using the DIC technique during the experiment (Section 3.1).

The results in Figure 4(b) indicate that the contact at the specimen-grip interface is realistically modeled with a friction coefficient of f = 0.1. In addition, this loading condition, with all three above sets of parameters, resulted in sufficiently close strain values to the ones calculated from Equation (4a) despite the relatively large lateral pressure. This is because the axial strain component is dominant with respect to the lateral ones because the epoxy specimen is not constrained axially and deforms easily due to its relatively low elastic modulus.

### 4.2. Hydrostatic Compression: Bare Fiber

**Figure 5.**
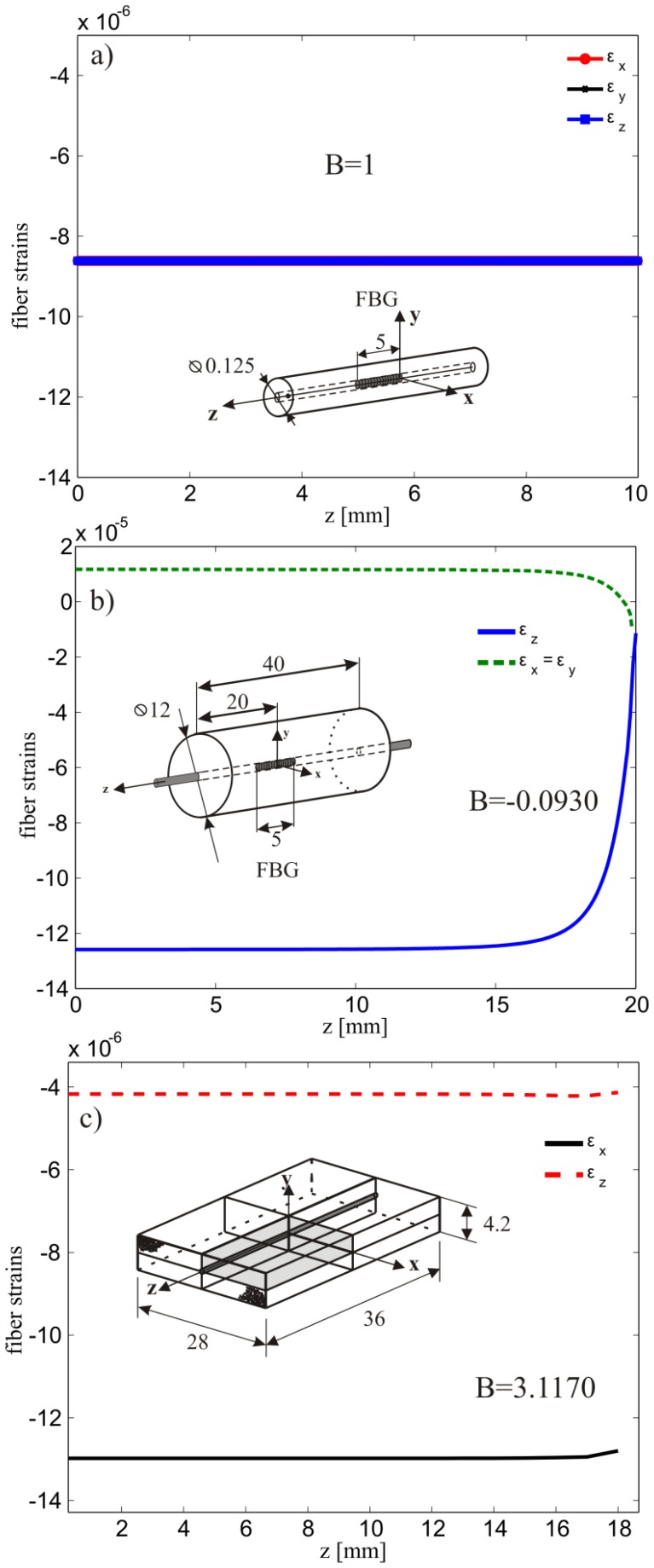
Simulated strains in the fiber core in the case of, (**a**) bare fiber, (**b**) embedded in an epoxy cylinder, (**c**) embedded in a composite. The pressure is 1 MPa and the units are mm.

**Figure 6.**
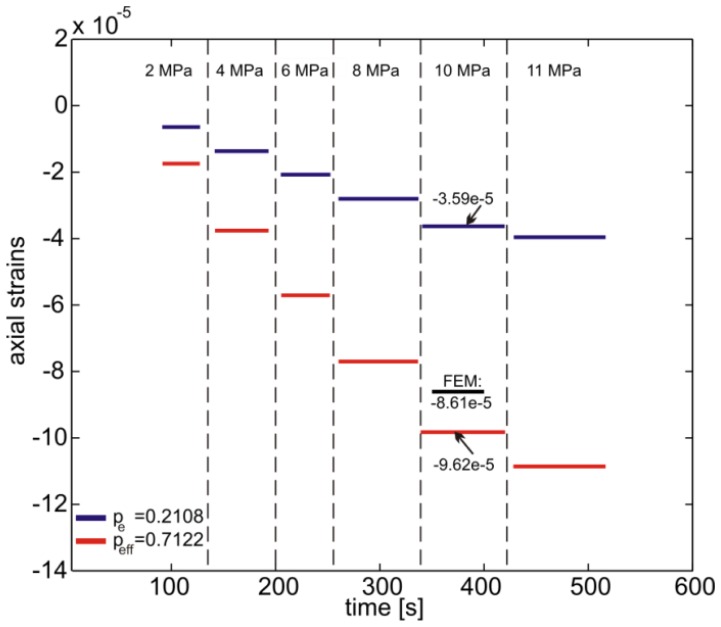
Bare fiber subjected to hydrostatic pressure: comparison between strain calculated from the wavelength shift measurements and Equation (4a) with *p_e_* = 0.2108 as well as Equation (6a) with $p_{e}^{\text{eff}}=0.7122$. The vertical dashed lines indicate the application time of each pressure step. The FE prediction for 10 MPa pressure is also shown.

The bare fiber was modeled as an isotropic linear elastic material with properties reported in Section 4.1. A uniform pressure of 1 MPa was applied to the external surface of the fiber and the axial and transversal deformations at the fiber axis were calculated. The results are reported in Figure 5(a) and as expected they are all equal with B = 1 corresponding to a $p_{e}^{\text{eff}}=0.7122$ calculated using Equation (6b) (see also Figure 2). The strain response, calculated from Equations (4a) and (6a), with the corresponding parameters *p_e_* and $p_{e}^{\text{eff}}$ and the experimental peak shift *Δλ_B_*, are reported in Figure 6 for different applied pressure levels.

In the same Figure the corresponding prediction of the FE model at a pressure of 10 MPa is also reported. Note that use of the corrected formulation (Equation 6a) dramatically reduces the error in the determination of the fiber axial deformation. In fact, the simplification introduced considering the lateral deformations equal to −*ν_f_ε_z_* generate an underestimation of the deformation by ∼170%. The ∼10% difference between the simulated strains and the ones from Equation (6a) is attributed firstly to an uncertainty in material properties of the glass fiber and secondly to random instrumentation noise.

Interestingly, if only the lateral surface is loaded and the circular sections are free, the numerical simulations give B= −2.1310 and Equation (6b) $p_{e}^{\text{eff}}=-0.6069$ which indicates that since the axial strain is not dominant, Equation (4a) is not appropriate. Comparing this case with that of the epoxy block, described in Section 4.1, the main difference here is the much greater elastic modulus of the glass which limits the axial strain in the core.

### 4.3. Hydrostatic Compression: Epoxy Cylinder

Taking advantage of the specimen symmetry only one half of the cylinder was modeled with ∼6600 axisymmetric quadratic elements with reduced integration. The domain was divided in two regions in order to model explicitly the presence the fiber along the specimen *z*-axis. In addition, perfect adhesion between the epoxy matrix and the fiber was imposed and the material properties used are the ones given in Section 4.1. On the entire surface of the specimen, a constant pressure of 1MPa was applied and the evolution of the axial and lateral deformation of the embedded fiber was calculated along the *z*-axis (Figure 5(b)). The FBG was centrally placed with respect to the specimen's symmetry plane where an extensive strain plateau is noticeable. In this region the calculated biaxiality ratio and effective photoelastic coefficient were *B* = −0.0930 and $p_{e}^{\text{eff}}=0.2515$, respectively. This latter value is used to compute the axial deformation of the fiber as function of the applied pressure.

**Figure 7.**
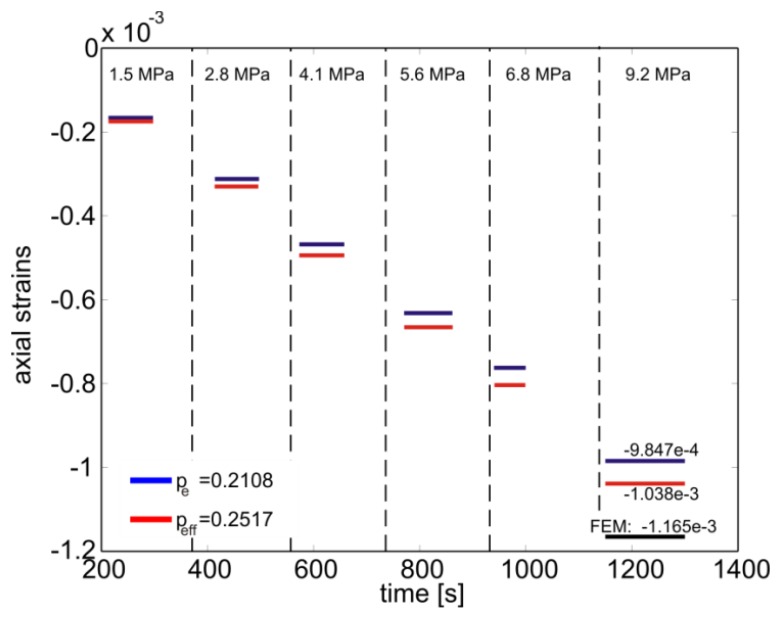
Epoxy cylinder subjected to hydrostatic pressure: comparison between strain calculated from the wavelength measurements and with Equation (4a) and *p_e_* = 0.2108 as well as Equation (6a) and $p_{e}^{\text{eff}}=0.2515$. The vertical dashed lines indicate the application time of each pressure step. The FE prediction for 9.2 MPa pressure is also shown.

The comparison of these calculations with the results given by Equation (4a) is reported in Figure 7 showing that little improvement is introduced by the Equation (6a) with respect to Equation (4a). Moreover, if only the pressure on the lateral surface is accounted for (*i.e.*, the pressure on circular sections of the specimen is set equal to zero), the simulations result in B = −0.2200 and $p_{e}^{\text{eff}}=0.1982$ from Equation (6b). Interestingly this value is practically equal to *p_e_* = 0.2108 (*i.e.*, 5.62% lower) and is explained from the fact that since there are no lateral constrains, the material is easily deformed along the axial direction. Thus the axial strain is dominant and reduces the effects of the lateral strains. This is similar to the loading case of the epoxy block in biaxial compression where the effects of the lateral strains on the calculated value of $p_{e}^{\text{eff}}$ were found negligible. Note also that the ∼10% difference between the simulated strains and the ones from Equation (6a) is attributed firstly to small uncertainties of elastic modulus of the epoxy under compression and secondly to random instrumentation noise.

### 4.4. Hydrostatic Compression: Unidirectional Composite

The unidirectional composite was modeled using ∼17,400 3D quadratic elements. The reinforcing fibers were aligned along the *z* direction and the material properties are reported in Table 1 [17].

One eighth of the specimen was modeled due to symmetry conditions with respect to the *xy, xz* and *yz* planes (Figure 5(c)). The external surfaces were loaded with a constant pressure of 1MPa and the corresponding transversal and axial strains as function of the *z*-coordinate are reported in Figure 5(c). Note here the large difference in the lateral strain as compared to the axial one. This is due to the fact that the composite is much stiffer along the fiber direction (Table 1). In this case the biaxiality ratio reaches the value of *B* = 3.117 leading to a $p_{e}^{\text{eff}}=1.6040$ (Equation 6b).

**Figure 8.**
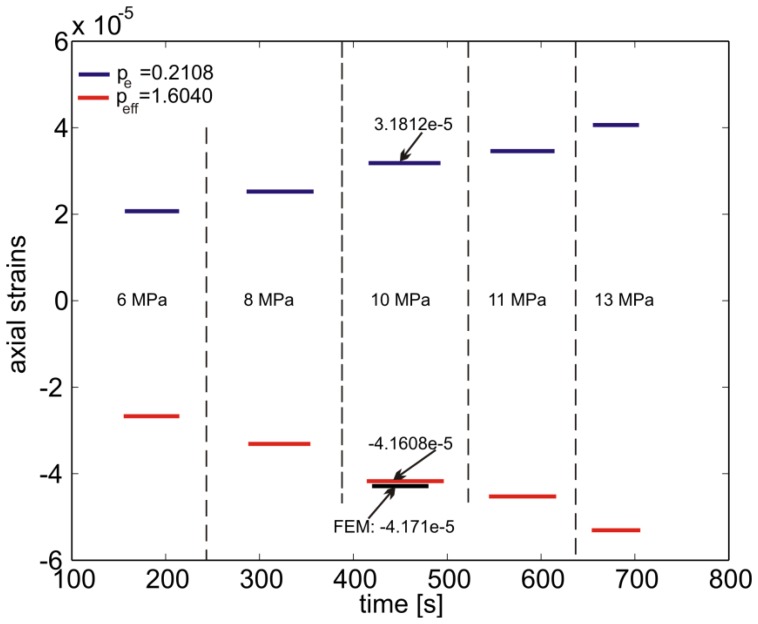
Unidirectional composite subjected to hydrostatic pressure: comparison between strain calculated from the wavelength measurements and Equation (4a) with *p_e_* = 0.2108 as well as Equation (6a) with $p_{e}^{\text{eff}}=1.6040$. The vertical dashed lines indicate the application time of each pressure step. The FE prediction for 10MPa pressure is also shown.

The fiber axial deformation calculated from the wavelength recorded during the experiment is shown in Figure 8. One can notice that Equation (4a) predicts a positive deformation of the composite material that is not physically possible under hydrostatic pressure conditions. On the contrary, the axial deformation computed by accounting for the actual strain environment of the fiber is well captured. In fact, in this condition the lateral strains dominate and, recalling Figure 2, such a high biaxiality ratio belongs to the negative branch of the normalized deformation equation (Equation 7) explaining the change in sign of the deformation. It should be noted that the results of the simulations are practically the same if the pressure is applied on the *yz* and *xz* planes (Figure 5(c)). This is explained from the fact that, (a) the area of the other two sides (*i.e.*, *xy* planes) of the specimen is considerably smaller than the rest of the specimen's area which results in a comparatively small applied force on the specimen, (b) the modulus of the composite is more than one order of magnitude greater in the *z* direction as compare to the transverse one (Table 1) which limits the axial deformation significantly. This is also validated by the numerical simulations. The nearly identical experimental and numerical results are due to the fact that the material properties of the composite are obtained by an identification technique of the material used [17].

## 5. Conclusions

The results of the combined experimental numerical analysis presented in this report demonstrate that the wavelength shift-strain relation (Equation 4a) from embedded FBG sensors gives accurate results only in cases where the axial strain dominates. This could be the case of an isotropic host material with relatively low modulus (as compared to that of the fiber). However, when the isotropic host material is of high modulus, use of the conventional optomechanics constant *p_e_* in Equation (4a) may not be realistic. Instead, an effective parameter $p_{e}^{\text{eff}}$ should be introduced whose values can be obtained from numerical simulations of the experimental setup. The results of this work also demonstrate that use of Equation (4a) can result in erroneous data when the host material is anisotropic as in the case of a composite material with the optical fiber along the reinforcing fibers. Due to the large elastic modulus along this direction, as compared to the transversal ones, the axial deformation is limited and the lateral strains become dominant. Thus the interpretation of *p_e_* in Equation (4a) is no longer valid. Instead an effective one should be introduced whose values can be calculated numerically using the properties of the host material. In practice, such a situation arises during processing of a composite reinforced with carbon fibers. Since these fibers have almost zero coefficient of thermal expansion, the axial strains during processing are very small as compared to the transversal ones.

Regarding the choice of the material parameters, in particular the strain-optic coefficients and refractive index of the fiber, it should be noted that for another set of these coefficients, *p*_11_ = 0.113, *p*_12_ = 0.252 [14] and *n_eff_* = 1.450, the vertical asymptote in Figure 2, changes its position slightly. As a consequence, the two parts of the curve towards the vertical asymptote (*i.e.*, for vales of *B* in the range of 0.5 and 5) give different results for $p_{e}^{\text{eff}}$ as compared with the results when *p*_11_ = 0.121, *p*_12_ = 0.270 [14] and *n_eff_* = 1.468. However, as long as the same coefficients are used to compare the results of the different experimental conditions, the conclusions on the importance of the lateral strains as compared to the axial one do not change.

## Figures and Tables

**Table 1.** Composite elastic properties.

**Elastic Property**								
E_zz_ [GPa]	E_xx_ [GPa]	E_yy_ [GPa]	ν_zx_	ν_zy_	ν_xy_	G_zx_ [GPa]	G_zy_ [GPa]	G_xy_ [GPa]
96.0	8.7	8.7	0.30	0.33	0.38	4.0	3.6	2.2
